# Yiwei decoction promotes apoptosis of gastric cancer cells through spleen-derived exosomes

**DOI:** 10.3389/fphar.2023.1144955

**Published:** 2023-06-01

**Authors:** Yingzhi Chen, Yu Li, Yue Wu, Shiyong Chen, Xiaoming Jin, Xuan Chen, Baoying Fei, Xiaomin Xue, Renzhao Wu, Kequn Chai

**Affiliations:** ^1^ School of Basic Medical Sciences, Zhejiang Chinese Medical University, Hangzhou, China; ^2^ Zhejiang Academy of Traditional Chinese Medicine, Hangzhou, China; ^3^ The First School of Clinical Medicine, Zhejiang Chinese Medical University, Hangzhou, China; ^4^ Stark Neuroscience Research Institute and Department of Anatomy, Cell Biology and Physiology, Indiana University School of Medicine, Indianapolis, IN, United States

**Keywords:** yiwei decoction (YWD), traditional chinese medicine (TCM), gastric cancer HGC-27 cells, apoptosis, tumour, spleen-derived exosomes

## Abstract

Yiwei decoction (YWD) is a formula of traditional Chinese medicine (TCM) that is clinically effective for the prevention and treatment of gastric cancer recurrence and metastasis. According to the theory of TCM, YWD tonifies the body and strengthens the body’s resistance to gastric cancer recurrence and metastasis potentially via the immune regulation of the spleen. The aims of the present study were to investigate whether YWD-treated spleen-derived exosomes in rats inhibit the proliferation of tumor cells, to elucidate the anticancer effects of YWD, and to provide evidence supporting the use of YWD as a new clinical treatment for gastric cancer. Spleen-derived exosomes were obtained by ultracentrifugation and identified by transmission electron microscopy, nanoparticle tracking analysis, and western blot analysis. The location of the exosomes in tumor cells was then determined by immunofluorescence staining. After tumor cells were treated with different concentrations of exosomes, the effect of exosomes on cell proliferation was determined by cell counting kit 8 (CCK8) and colony formation assays. Tumor cell apoptosis was detected by flow cytometry. Particle analysis and western blot analysis identified the material extracted from spleen tissue supernatant as exosomes. Immunofluorescence staining showed that spleen-derived exosomes were taken up by HGC-27 cells, and the CCK8 assay confirmed that the relative tumor inhibition rate of YWD-treated spleen-derived exosomes in the 30 μg/mL reached 70.78% compared to control exosomes in the 30 μg/mL (*p* < 0.05). Compared to control exosomes in the 30 μg/mL, the colony formation assay indicated that YWD-treated spleen-derived exosomes in the 30 μg/mL colonies have decreased by 99.03% (*p* < 0.01). Moreover, flow cytometry analysis showed that treatment with YWD-treated exosomes in the 30 μg/mL increased the apoptosis rate to 43.27%, which was significantly higher than that of the control group in the 30 μg/mL (25.91%) (*p* < 0.05). In conclusion, spleen-derived exosomes from YWD-treated animals inhibit the proliferation of HGC-27 cells via inducing apoptosis, suggesting that spleen-derived exosomes are involved in mediating the antitumor effect of YWD. These results demonstrated a novel exosome-mediated anticancer effect of YWD as a TCM formula, thereby supporting the use of YWD-treated exosomes as a new approach for the clinical treatment of gastric cancer.

## Introduction

The incidence and mortality of cancer are rapidly increasing worldwide (Sung et al., 2021). An estimated more than 4.56 million newly diagnosed cancer cases and more than 3.00 million cancer deaths occurred in China in 2020. 38.8% of all cancer cases diagnosed in China were digestive cancers (including colorectal cancer, stomach cancer, liver cancer, and esophageal cancer) in 2020 (Qiu et al., 2021). Gastric cancer is a high incidence tumor. Due to the aging population and the increase in high-risk groups, these burden estimates will continue to increase.

At present, surgery is the only treatment that can completely eradicate gastric cancer (Johnston and Beckman, 2019; Smyth et al., 2020). After gastric tumors are removed, however, the cancer can easily relapse and metastasize, resulting in poor prognosis and reduction of quality of life (Qiu et al., 2022). Most early gastric cancers are asymptomatic when diagnosed (Gullo et al., 2020), but once diagnosed, they are terminal. Studies have shown that traditional Chinese medicine (TCM) plays an active role in the prevention and treatment of gastric cancer, especially in improving clinical symptoms (Yang et al., 2021). Yiwei decoction (YWD) is an empirical prescription of Traditional Chinese medicine (TCM) that has been used for the treatment of gastric cancer in TCM clinical practice.

Exosomes are microvesicles and are essential for intercellular communication (Zhang and Yu, 2019). Exosomes are polymorphic vesicle-like bodies secreted by various cell types, and they originate from late endosomes (or polycystic endosomes) of the endocytic system. Under electron microscopy, exosomes are mostly 30–150 nm in diameter with flat, spherical, or cup-shaped shapes, and they are enclosed in phospholipid bilayers (Ning et al., 2018). Exosomes not only play an important role in tumorigenesis, growth, and metastasis (Wang et al., 2014; Huang et al., 2020; Jia et al., 2020; Xu et al., 2020) but also have inhibitory effects on a variety of cancers, including gastric cancer (Fu et al., 2019), hepatocellular carcinoma (Li et al., 2020), non-small cell carcinoma (Viaud et al., 2009; Besse et al., 2016), and breast cancer (Shi et al., 2020). Exosomes isolated from myeloid-derived suppressor cells (MDSCs) extracted from the spleen have intermediate immunosuppressive capacity compared to exosomes isolated from MDSCs in the primary tumor region or bone marrow (Rashid et al., 2021). Reducing CD19^+^ exosomes from B Cells in the spleen has the potential to improve the antitumor effects of chemotherapy (Zhang et al., 2019). Exosomes derived from bone marrow mesenchymal stem cells (BM-MSC) combined with anti-tumor drugs have achieved remarkable therapeutic effects in cancer treatment (Zhou et al., 2021). However, to the best of our knowledge, no studies have reported whether exosomes from the spleen after any other drug intervention enhance antitumor effects.

At present, the existing literature studies have reported the antitumor effects of exosomes produced by single cells, such as splenic T Cells and B Cells (Zhang et al., 2019; Rashid et al., 2021). As a traditional Chinese herbal medicine formula, YWD achieves an antitumor effect by improving the body’s resistance to cancer, of which the mode of action is likely multitargeted and involves multiple pathways. Compared to single cells, such as T Cells and B Cells, derived from the spleen, YWD may affect exosomes produced by multiple cells in the spleen to improve tumor immunity through a multitargeted and multipathway effect.

YWD is an herbal formula used for chronic prevention and treatment of gastric cancer recurrence and metastasis. The formula consists of *Astragalus membranaceus*, *Pinellia ternate*, *Tetrastigma hemsleyanum Diels et Gilg*, *Actinidia chinensis Planch*, *Ophiopogon japonicus*, *Taraxacum mongolicum Hand*, *Paeonia lactiflora Pall*, *Coix lacryma-jobi L.var.ma-yuen (Roman.) Stapf*, and *Rabdosia amethstoides (Benth) Hara*. Because the effect of YWD is characterized by supplementing qi and strengthening the body (enhancing the body’s resistance to diseases), it may be related to the function of immune regulation of the spleen. Therefore, further research into the antitumor effects of spleen-derived exosomes is a reasonable research direction. In this study, we hypothesized that spleen-derived exosomes with or without YWD could enter tumor cells and promote tumor cell apoptosis when co-cultured with tumor cells. The spleen-derived exosomes of rats treated with YWD have a more significant anti-tumor effect on tumor cells than the same exosomes without YWD.

The main purpose of the present study was to investigate the anticancer effects of YWD-treated spleen-derived exosomes and to provide evidence for their use as a new strategy for the clinical treatment of gastric cancer.

In the present study, control and YWD-treated spleen-derived exosomes isolated from the supernatant of rat spleen tissue were used to evaluate the apoptosis effect of YWD on human gastric cancer cells (HGC-27 cells), murine forestomach carcinoma (MFC cells), and mouse malignant sarcoma cells (S180 cells).

## Materials and methods

### Experimental herbs

*Astragalus Membranaceus (Inner Mongolia, China)*, *Pinellia Ternate (Zhejiang, China)*, *Tetrastigma Hemsleyanum Diels et Gilg (Zhejiang, China)*, *Actinidia chinensis Planch (Zhejiang, China)*, *Ophiopogon Japonicus (Zhejiang, China)*, *Taraxacum mongolicum Hand (Shandong, China)*, *Paeonia lactiflora Pall (Zhejiang, China), Coix lacryma-jobi L.var.ma-yuen (Roman.) Stapf (Zhejiang, China)*, and *Rabdosia amethstoides (Benth.) Hara (Zhejiang, China)* were purchased from Hangzhou Huadong Chinese Medicine Beverage Co. and appraised by Zhongming Yu, Deputy Director of Zhejiang Institute of Traditional Chinese Medicine to conform to China Pharmacopoeia (2020 edition) or Zhejiang Chinese Medicine Processing Standard (2015 edition) (Table 1).

**TABLE 1:** Catalogue of information related to the source of experimental medicinal materials for the YWD [China Pharmacopoeia (2020 edition); Zhejiang Chinese Medicine Processing Standard (2015 edition)].

Latin name	Access number	Place of production	Source	China national medical insurance drug code
Astragalus membranaceus	170824	Inner Mongolia, China	Dried roots	T001700368
Pinellia Ternate	170804	Zhejiang, China	Dried tuber	T001300390
Tetrastigma Hemsleyanum Diels et Gilg	170830	Zhejiang, China	Dried tuber	T330203592
Actinidia chinensis Planch	170819	Zhejiang, China	Dried root	T330201963
Ophiopogon japonicus	170818	Zhejiang, China	Dried tuber	T001700535
Taraxacum mongolicum Hand	170901	Shandong, China	Dried tuber	T000200610
Paeonia lactiflora Pall	170715	Zhejiang, China	Dried root	T331705979
Coix lacryma-jobi L.var.ma-yuen (Roman.) Stapf	170723	Zhejiang, China	Dried mature seeds	T000600852
Rabdosia amethstoides (Benth.) Hara	170823	Zhejiang, China	Dried rhizome	T330203598

### Preparation of aqueous extracts of YWD

We mixed 300 g of *Astragalus Membranaceus*, 120 g of *Pinellia ternate*, 180 g of *Tetrastigma Hemsleyanum Diels et Gilg*, 300 g of *Actinidia chinensis Planch*, 150 g of *Ophiopogon Japonicus*, 210 g of *Taraxacum mongolicum Hand*, 120 g of *Paeonia lactiflora Pall*, 300 g of *Coix lacryma-jobi* L.var.ma-yuen (Roman.) Stapf, and 300 g of Rabdosia ameths*toides (Benth) Hara*. After adding 10 times the amount of water to the mixture, the mixture was incubated for 30 min. An extract was then obtained by heating (105°C) and refluxing three times (2 h each time). The extract filtrate was combined and concentrated to obtain an YWD extract with a concentration of 1.09 g/mL (Dong et al., 2020).

### Grouping and administration of rats in preparation of serum and spleen-derived exosomes for YWD

Twelve 12-week-old Wistar rats were obtained from the Hangzhou Medical College [Laboratory Animal Production License No. SCXK (Zhe) 2019–0002 and Certificate of Conformity No. 20210813Abab0100018988] and used for the pilot experiment, and 24 10-week-old Wistar rats were obtained from Shanghai Slack Experimental Animal Co., Ltd. [Experimental Animal Production License No. SCXK (Shanghai) 2022–0004 and Certificate No. 20220004017522] were used in the subsequent experiment. All procedures were approved by the Experimental Animal Welfare Ethics Committee of Zhejiang Institute of Traditional Chinese Medicine [Approval No. ZCRI Animal Ethics Review Nos. 021 (2019) and 038 (2021)] and were performed according to the published Guidelines for the Care and Use of Laboratory Animals, National Institutes of Health (NIH Publications, No. 8023, revised 1978). The rats were divided into low, medium, and high dose gavage groups (5 g/kg, 10 g/kg, and 20 g/kg, respectively) and a control group (6 animals in each group). After the rats were gavaged for 3 days, they were anesthetized with 10% chloral hydrate solution (0.3 mL/100 g, i.p.) at 3 h after gavage on the third day. The serum of low, medium and high doses was taken out for the pre-experiment of selecting the optimal dose of tumor inhibition; After that, low, medium and high doses of spleens were taken out for extracting and separation of exosomes from spleen. Based on the results of the pilot experiment, an optimal YWD dosage of 20 g/kg was selected for the subsequent experiments.

### Pre-experiment of selecting the optimal dose of serum tumor inhibition

The drugs were administered by gavage as described above, and the blood of rats in the low, medium, and high dose groups (5 g/kg, 10 g/kg, and 20 g/kg, respectively) as well as the control group (only given drinking water) was collected. The supernatant was obtained by centrifugation for 10 min at 3500 r/min, and the obtained sera were named as low-dose YWD-treated serum, medium-dose YWD-treated serum, high-dose YWD-treated serum, and control serum (no YWD treatment). HGC-27 cells were cultured in RPMI 1640 medium (Cienry, China, Cat. No. CR31800-S) containing 10% fetal bovine serum at 37°C and 5% CO_2_. At the logarithmic growth phase of HGC-27 cells, cells were seeded into 24-well plates (1.5 × 10^4^ cells/well) and cultured for 24 h to ensure adherence of cells. Cells were then treated with 1% low-dose YWD-treated serum, 1% medium-dose YWD-treated serum, 1% high-dose YWD-treated serum, and 1% serum control serum for 48 h. Cells were then digested with trypsin and counted. The highest tumor inhibition rate was then determined for the optimal dose.

### Isolation and purification of exosomes

After euthanizing the rats, the spleens were removed, ground, filtered in 10 mL of RPMI 1640 medium. The filtered liquid was centrifuged at 4°C (300 × g for 10 min, 2500 × g for 15 min, and 10000 × g for 30 min) before being filtered using a 0.22 μm capsule filter (Millipore, Billerica, MA, United States). The liquid was added to an ultracentrifuge tube with the lower layer containing 5 mL of 30% heavy water sucrose cushion (Beckman, Germany) (in order to embed exosomes into the heavy water sucrose cushion and achieve the purpose of purifying exosomes) to form a visible interphase. After 70 min of ultracentrifugation at 120000 × g and 4°C using a SW-32Ti pendulum rotor (Optima L-90K, Beckman Coulter, Germany), the sucrose cushion containing exosomes were collected from the bottom of the tube and transferred to a new tube. 1xPBS (Cienry, Zhejiang, China, Cat. No. CR20012) was then added followed by ultracentrifugation at 120000×*g* and 4°C for 70 min. The precipitate was collected and filtered through a 0.45 μm capsule filter before being stored at −70°C (Zhang et al., 2020; Wang et al., 2021). Protein concentrations were determined using a bicinchoninic acid (BCA) kit (Beyotime, Shanghai, China, Cat. No. P0012).

### Transmission electron microscopy of exosome morphology

In order to verify that the spleen-derived exosomes conform to the expected appearance, transmission electron microscopy (TEM) was performed. The purified exosome solution (20 μL) was uniformly placed on a Formvar-carbon copper mesh sample carrier with a diameter of 2 nm. Following incubation for 1 min at room temperature, the exosome solution was gently aspirated from the edge of the copper mesh with filter paper. Next, a 3% (w/v) phosphotungstic acid solution (pH 6.8) (Ted Pella Inc., CA, United States) was added to the copper mesh and negatively stained for 1 min at room temperature. Finally, the copper mesh was dried under incandescent light and placed into the sample chamber of a transmission electron microscope (Hitachi H7650 TEM, Tokyo, Japan) to observe and photograph exosome morphology.

### Nanoparticle tracking analysis of exosome size

In order to find out whether the diameter of the extracted spleen exosomes was consistent with the size of exosomes, nanoparticle tracing analysis was performed. Sample cells were washed with deionized water, and the NanoSight NS 300 (Malvern Panalytical, United Kingdom) instrument was calibrated with polystyrene microspheres (110 nm). The sample cells were then washed with PBS (Biological Industries, Israel) and diluted with PBS at a ratio of 1:30000 and injected for detection.

### Western blot assay

In order to know whether the extracted spleen exosomes have the characteristic proteins of exosomes, the characteristic proteins of the above three gavage doses of rats were identified to know the repeatability of the three doses of characteristic proteins. Western blot analysis was used to detect exosome surface markers, such as tumor susceptibility gene 101 protein (anti-TSG101) (Abcam, Cambridge, MA, United States, Cat. No. AB83, molecular weight: 47 KD), recombinant tetraspanin 30 cluster of differentiation 63 (anti-CD63; Abcam, Cambridge, MA, United States, Cat. No. AB193349, molecular weight: 26–48 KD), ALG-2-interacting protein X (anti-ALIX; Abcam, Cambridge, MA, United States, Cat. No. AB275377, molecular weight: 96 KD), and programmed cell death 6 (anti-ALG-2, also known as PDCD6; Protentech, United States, Cat. No. 12303-1-AP, molecular weight: 22 KD), glyceraldehyde-3-phosphate dehydrogenase (anti-GAPDH; Diagbio, Hangzhou, China, Cat. No. db106, molecular weight: 36 KD) as internal reference protein. More than three of the four proteins mentioned above need to be positive to meet the requirements of exosome characteristic proteins (Mahul-Mellier et al., 2009; Thery et al., 2018). After the BCA protein concentration was quantified (Beyotime, Shanghai, China), the proteins were separated by sodium dodecyl sulfate-polyacrylamide gel electrophoresis (SDS-PAGE) using the following gels: TSG101 and CD-63 (10 µg loaded per lane) were separated by 10% SDS-PAGE and 5% concentrated gel; ALG-2 (0.775 µg loaded per lane) was separated by 12% SDS-PAGE and 5% concentrated gel; and ALIX (0.775 µg loaded per lane) was separated by 8% SDS-PAGE and 5% concentrated gel. The proteins were transferred to polyvinylidene difluoride (PVDF) membranes (Immobilon-P, United States) under standard wet transfer conditions. The PVDF membranes were blocked with TBST solution containing 10% skim milk (Beyotime, China) at room temperature for 1 h. After four washes with PBST (8 min each wash), the PVDF membranes were incubated with the following primary antibodies overnight at 4°C: anti-CD63, anti-TSG101, anti-ALG-2, anti-ALIX, and anti-GAPDH antibodies (all 1: 1000 dilution). After four washes with PBST (8 min each wash), the PVDF membranes were incubated with horseradish peroxidase (HRP)-conjugated secondary antibodies (Abcam, Cambridge, MA, United States, Cat. No. AB205719) at room temperature for 1 h. After four washes with PBST (8 min each wash), the membranes were developed using Immobilon Western HRP luminescent reagent (Millipore, United States), and the results were analyzed.

### Immunofluorescence staining of exosomes entering tumor cells

In order to know whether the spleen exosomes added into the co-culture of tumor cells could enter the tumor cells, immunofluorescence staining was performed. Cells (0.75 × 10^4^ cells/well) were seeded into 24-well glass coverslips (500 µL of culture medium/well). Four groups were set up, with 3 holes in each group [HGC-27 cells without exosome treatment group, spleen-derived exosomes (no HGC-27 cells) group, HGC-27 cells treated with control spleen-derived exosomes, HGC-27 cells treated with YWD-treated spleen-derived exosomes.]. Once the cells reached an appropriate density (approximately 80% confluence on the coverslip), the exosomes were labeled with PKH67 (Umibio, China, Cat. No. UR52303) according to the manufacturer’s instructions. In brief, a PKH67 staining working solution (10 µL) was prepared by mixing the “PKH67” and “Dilution C” solutions in a 1:9 ratio, and 990 µL of PBS was added to obtain 1000 µL of PKH67 staining working solution. Then, 10 µL of staining solution was added to the exosomes (10 µg of control spleen-derived exosomes and 10 µg of YWD-treated spleen-derived exosomes, respectively) and shaken for 1 min, keeping still for 30 min. The mixture was then added to the cover glass containing HGC-27 cells. After incubation in the staining solution for 17–24 h, cells were washed three times with PBS and fixed with 4% paraformaldehyde for 15 min. Cells were then washed three times with PBS, permeabilized with 0.4% Triton™ X-100 (Vetec™, Shanghai, China), washed three times with PBS, and incubated with Acti-stain™ 555 Rhodamine Phalloidin (Cytoskeleton, Denver, CO, United States, Cat. No. CA1610) for 1 h to stain the cytoplasm. Cells were then washed five times with PBS and incubated with 1 g/mL 4′,6-diamidino-2-phenylindole (DAPI) (Coolaber, Beijing, China, Cat. No. SL7100) to stain the nuclei. Finally, images were obtained using a confocal laser scanning microscope (Zeiss LSM 800, Germany).

### Effect of YWD-treated exosomes on cell proliferation

In order to understand whether spleen exosomes with or without YWD have different anti-tumor effects on tumor cells in co-culture, Cell Counting Kit 8 (CCK8) (Dojindo, Tokyo, Japan, Cat. No. 10011018) experiment was performed. HGC-27 cells (purchased from National Collection of Authenticated Cell Cultures, China, Cat. No. TCHu22, MFC cells (purchased from Conservation Genetics CAS Kunming Cell Bank, China, Cat. No. KCB92020YJ, and S180 cells (purchased from Conservation Genetics CAS Kunming Cell Bank, China, Cat. No. KCB93029YJ were cultured in RPMI 1640 medium containing 10% fetal bovine serum at 37°C and 5% CO_2_. During the logarithmic growth phase, cells were seeded into 96-well plates (5 × 10^3^ cells/well) (NEST, Wuxi, China) and cultured for 24 h to ensure cell adherence. Cells were then treated with 6.67 μg/mL paclitaxel (Haikou Pharmaceutical Factory Co., Ltd., China, Cat. No. 12171202; positive control group) and different concentrations of YWD-treated exosomes (7.5 μg/mL YWD-treated group, 30 μg/mL YWD-treated group, and 120 μg/mL YWD-treated group) and YWD-free exosomes (untreated spleen-derived exosomes; 7.5 μg/mL control exosomes, 30 μg/mL control exosomes, and 120 μg/mL control exosomes). Three to four parallel wells were treated for each concentration. Cells treated only with RPMI 1640 medium were used as the tumor cell control group (model group). After the treatments were completed, 10 µL of CCK8 was added to each well followed by incubation for 3 h in the dark. After gentle shaking, the absorbance (A450 nm) values were measured with an enzyme marker (BioTek, Shanghai, China). The tumor cell tumor suppression rates were then calculated using the following two formulas: 1) tumor suppression rate relative to the tumor cell model group (%) = (model group - experimental group)/model group x 100%; and 2) relative tumor suppression rate compared to the control tumor cell exosomes (%) = (control exosomes—YWD-treated exosomes)/control exosomes x 100%

### Colony formation assay

In order to understand whether the spleen exosomes with or without YWD have different anti-tumor effects on HGC-27 tumor cells in co-culture, colony formation assay was performed. HGC-27 cells were seeded at a density of 1000 cells/well in 24-well plates. After 14 days, the culture was finished until cell clusters appeared. Different concentrations of exosomes (7.5 μg/mL and 30 μg/mL) and paclitaxel (positive control group, 6.67 μg/mL) were added to the treatment group, After 24 h, cells were washed three times with PBS, fixed with 4% paraformaldehyde, and stained with 0.1% crystal violet. Using ImageJ analysis software (Chi et al., 2022), we selected colonies in the range of 50–1000 pixels under the threshold value of 74, and we counted the number of colonies. The colony formation rate was calculated using the following formula: colony formation rate (%) = number of colonies/inoculum (1000) x 100% (Lin et al., 2021; Dong et al., 2022).

### Apoptosis assay

In order to find out whether spleen exosomes with or without YWD have different anti-tumor and pro-apoptotic effects on tumor cell line HGC-27 when co-cultured, apoptosis assay was performed. HCG-27 cells were cultured in 6-well plates (2 × 10^5^ cells/well) (NEST, Wuxi, China) with exosomes (7.5, 30, and 120 μg/mL) or paclitaxel (positive control group; 6.67 μg/mL) for 48 h. Cells were then washed twice with PBS, digested with trypsin (Cienry, China, Cat. No. CR27250), and collected by centrifugation at 1000 r/min for 5 min (centrifugal radius; r = 21 cm). Cells were then resuspended at a concentration of 1 × 10^6^ cells/mL, and apoptosis was measured using an apoptosis kit (Cwbiotech, Beijing, China, Cat. No. CW25745). After adding dye-free or fluorescein isothiocyanate-labeled Annexin V/propidium iodide (*i.e.*, Annexin V-FITC/PI) to 100 µL of cell suspension in flow tubes (including four flow tubes for the control group), the cells were mixed and incubated for 15 min at room temperature in the dark. Then, 400 µL of PBS was added to each tube and mixed well, and the apoptotic cells were analyzed by flow cytometry (Becton Dickinson, Franklin Lakes, NJ, United States). For each analysis, 10,000 events were evaluated with FlowJo 7.6 software (Becton Dickinson, Franklin Lakes, NJ, United States).

### Statistical analysis

SPSS 25.0 statistical software (Chicago, IL, United States) was used for the statistical analyses.The data are presented as the mean ± standard deviation. If the data were normally distributed, the Shapiro-Wilk test was utilized. Levene’s test was used to evaluate the variance. The Bonferroni test was used for homogeneous variance, and the Games-Howell test was used for heterogeneous variance. If the data were non-normally distributed, the Kruskal-Wallis test was used. On the basis of the above tests, the three concentrations of spleen-derived exosomes (7.5 μg/mL, 30 μg/mL, and 120 μg/mL) were added as independent samples with drug treatment and without drug treatment. The *t*-test was used if the data showed normal distribution, and the Kruskal-Wallis test was used if the data were non-normally distributed. *p* < 0.01 or *p* < 0.05 was considered statistically significant.

## Results

Preliminary screening of the optimal dosage of gastric perfusion found that YWD at a dosage of 20 g/kg had an optimal tumor inhibition effect on HGC-27 cells (Table 2).

**TABLE 2:** Preliminary screening of the effects of low, medium, and high doses of rat serum containing YWD on HGC-27 cell growth.

Group	Cell population (unit: PCs/mL)	Tumor inhibition rate (%) (compared to the model group)	Relative tumor inhibition rate (%) (compared to the control serum group)
Model group	29.29 × 10^4^	/	/
Control serum group	27.91 × 10^4^	/	/
5 g/kg YWD-treated serum group	32.18 × 10^4^	0	0
10 g/kg YWD-treated serum group	9.08 × 10^4^	69.01%	68.49%
20 g/kg YWD-treated serum group	6.33 × 10^4^	78.40%	77.34%

### Isolation and identification of exosomes

**FIGURE 1:**
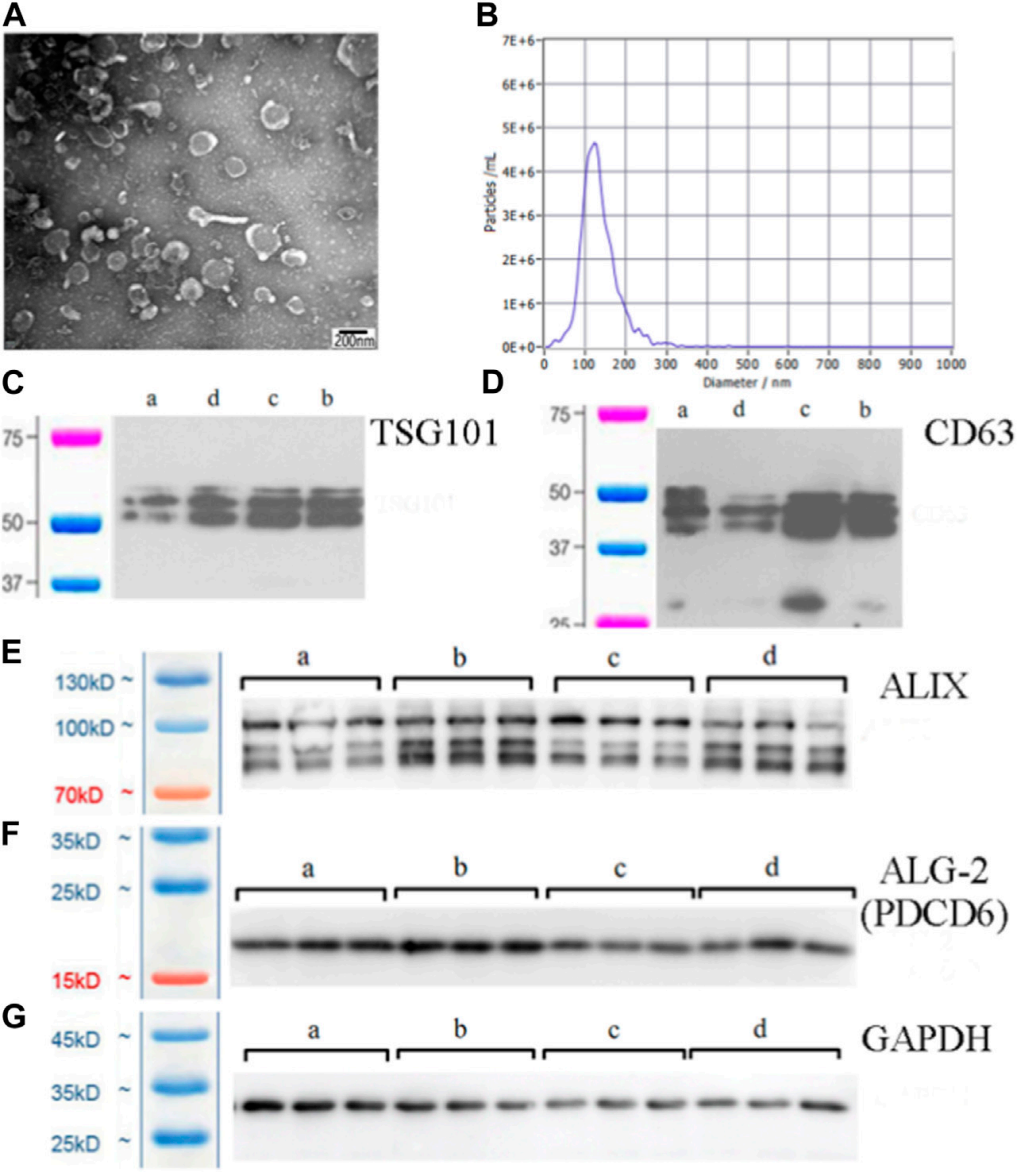
Exosomes purified from spleen tissue supernatant. **(A)** Morphology of exosomes under transmission electron microscopy (×60000 magnification) (scale bar = 200 nm). **(B)** Size distribution and agglutination of exosomes studied by nanoparticle tracking analysis. **(C)** Western blot analysis of the TSG101 exosome-specific marker. **(D)** Western blot analysis of the CD63 exosome-specific marker. **(E)** Western blot analysis of the ALIX exosome-specific marker. **(F)** Western blot analysis of the ALG-2 exosome-specific marker. The lanes are labelled as follows: a, control exosome group; b, low dose YWD-treated exosomes group; c, medium dose YWD-treated exosomes group; and d, high dose YWD-treated exosomes group. **(G)** GAPDH (internal reference protein). Note: Spleen-derived exosomes were extracted from rats after intragastric administration of low, middle, and high doses of YWD (5 g/kg, 10 g/kg, and 20 g/kg, respectively).

In the present study, we used transmission electron microscopy, nanoparticle tracking, and western blot analysis to identify exosomes. Transmission electron microscopy illustrated that the exosomes had a cup-shaped appearance (Figure 1A), which agreed with recent exosome studies (Villatoro et al., 2020). Nanoparticle tracking analysis indicated that the isolated exosomes were 100–150 nm in size (Figure 1B). The characterization of exosomes requires at least three positive indexes, such as TSG101, CD63, ALIX, and ALG-2 (Mahul-Mellier et al., 2009; Thery et al., 2018). We identified these four exosomes’ characteristic proteins, and the results were all positive. (Figures 1C–F).

### Exosome entry into HGC-27 cells

**FIGURE 2:**
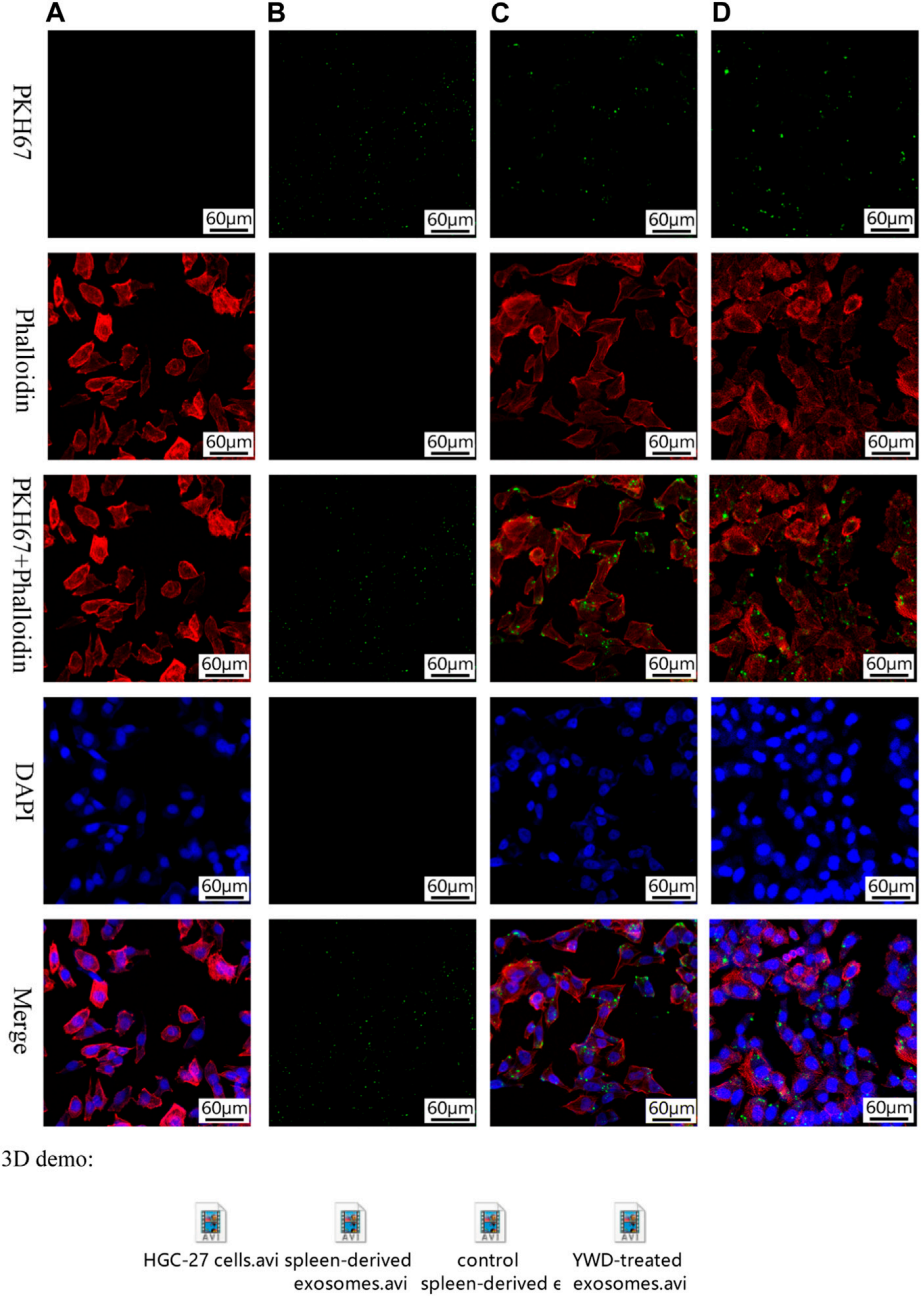
Fluorescence staining of exosomes taken up by HGC-27 cells (×200 magnification). PKH67 staining, phalloidin staining, PKH67+ phalloidin staining, DAPI staining, and merged images of **(A)** HGC-27 cells without exosomes treatment, **(B)** spleen-derived exosomes (no HGC-27 cells), **(C)** HGC-27 cells treated with control spleen-derived exosomes, and **(D)** HGC-27 cells treated with YWD-treated spleen-derived exosomes.

We used fluorescence staining to determine whether spleen-derived exosomes are taken up into HGC-27 cancer cells *in vitro*. PKH67, phalloidin, and DAPI staining detected splenic exosomes, cytoplasm, and nuclei, respectively, in HGC-27 cells exposed to YWD-treated exosomes or control exosomes (Figures 2A–D). The staining results indicated that exosomes extracted from spleen tissue were taken up by HGC-27 cells.

### YWD-treated exosomes affect tumor cell viability

Because Chinese herbal compounds may have a good curative effect on gastric tumors in the clinic, we investigated the effects of spleen-derived control and YWD-treated exosomes on three cancer cell types *in vitro*. Compared to the model group (no treatment, cells treated only with RPMI 1640 medium were used as the tumor cell control group), the tumor inhibition rates of control spleen-derived exosomes at concentrations of 7.5 μg/mL, 30 μg/mL, and 120 μg/mL were 18.47%, 66.91%, and 90.80% in HGC-27 cells, respectively; at the same concentrations, the antitumor rates of YWD-treated spleen-derived exosomes were 30.32%, 90.33%, and 94.93%, in HGC-27 cells, respectively. In S180 cells, the tumor inhibition rates of the control spleen-derived exosomes at concentrations of 7.5 μg/mL, 30 μg/mL, and 120 μg/mL were 2.93%, 18.86%, and 79.99%, respectively, while the tumor inhibition rates of the YWD-treated spleen-derived exosomes were 40.57%, 46.41%, and 89.83%, respectively. In MFC cells, the tumor inhibition rates of the control spleen-derived exosomes were 2.99%, 34.29%, and 41.35% at concentrations of 7.5 μg/mL, 30 μg/mL, and 120 μg/mL, respectively, while the tumor inhibition rates of the YWD-treated spleen-derived exosomes were 8.12%, 46.46%, and 50.39%, respectively. Together, these results indicated that the YWD-treated spleen-derived exosomes have a good antitumor effect at the same concentrations compared to the model group.

**FIGURE 3:**
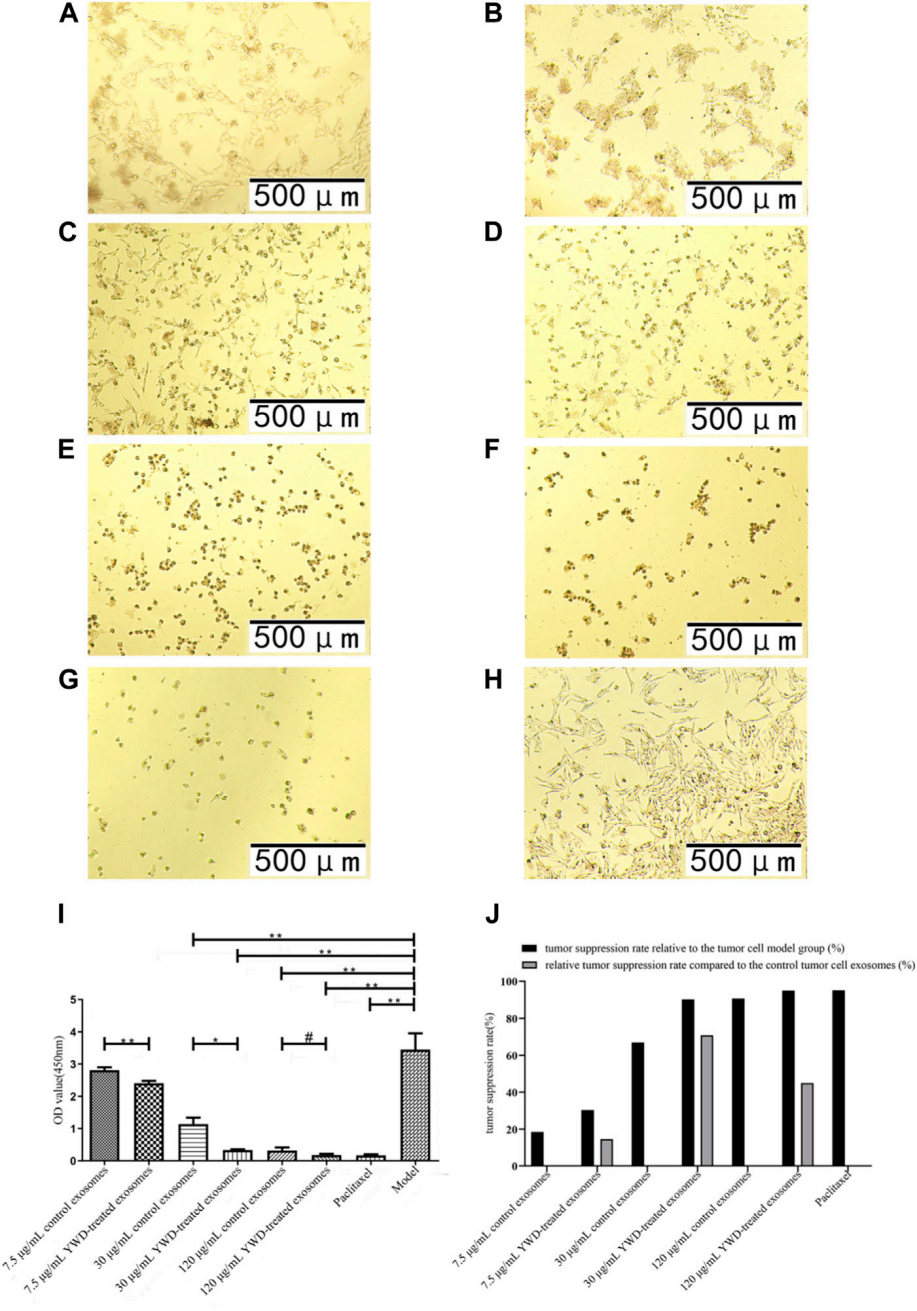
Effects of YWD-treated and control spleen-derived exosomes on the proliferation of HGC-27 cells (×100 magnification). CCK8 assays were performed to detect cell proliferation (*n* = 4) in the **(A)** 7.5 μg/mL control spleen-derived exosomes group. **(B)** 7.5 μg/mL YWD-treated spleen-derived exosomes group. **(C)** 30 μg/mL control spleen-derived exosomes group. **(D)** 30 μg/mL YWD-treated spleen-derived exosomes group. **(E)** 120 μg/mL control spleen-derived exosomes group. **(F)** 120 μg/mL YWD-treated spleen-derived exosomes group. **(G)** paclitaxel group. **(H)** model group (cells treated only with RPMI 1640 medium were used as the tumor cell control group). **(I)** The proliferation was determined via CCK8 assay and **(J)** tumor suppression rate. (Note: **p < 0.01 and *p < 0.05; #Independent sample *t*-test with and without YWD, indicating #*p* < 0.05).

**FIGURE 4:**
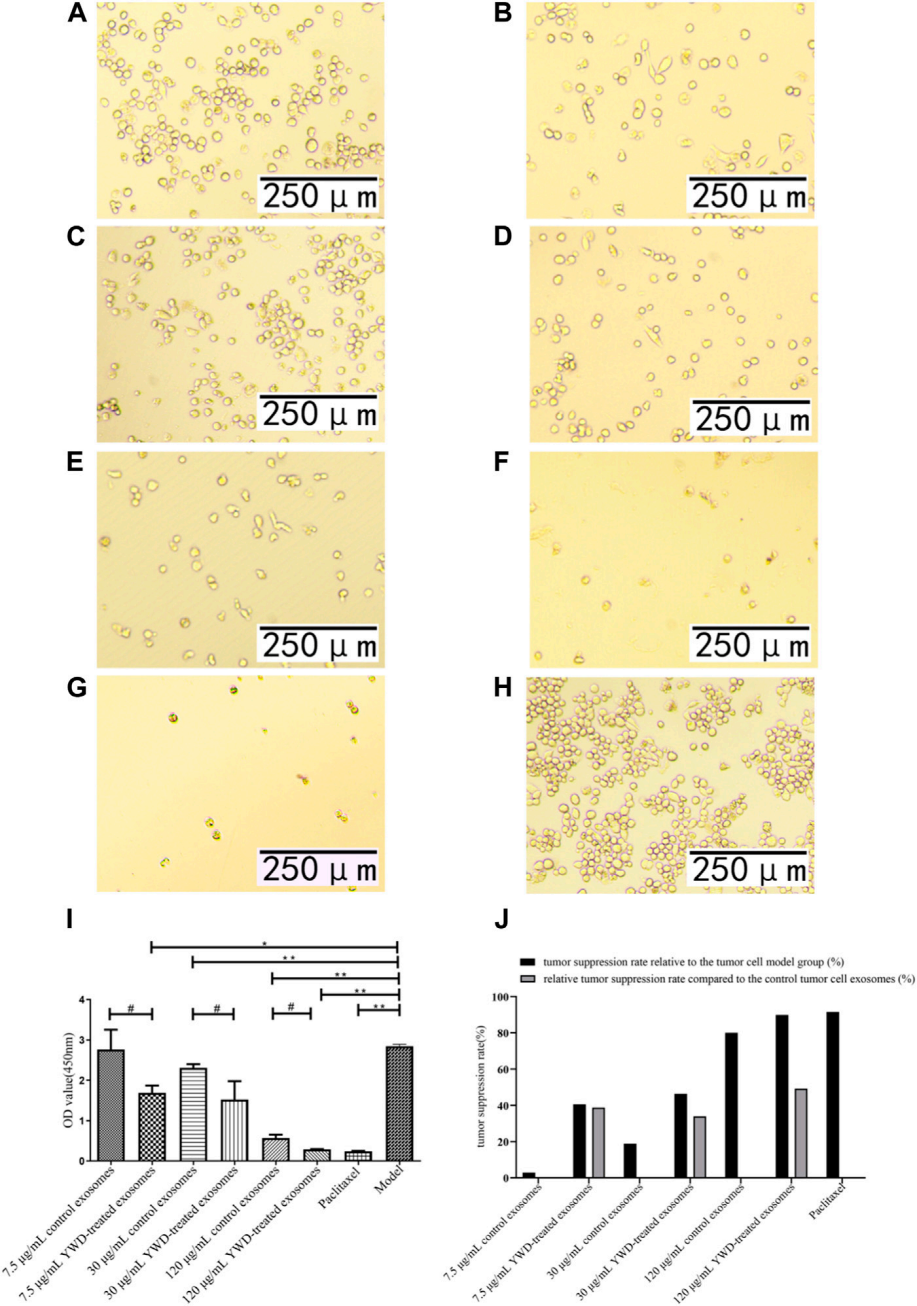
Effects of YWD-treated and control spleen-derived exosomes on the proliferation of S180 cells (×200 magnification). CCK8 assays were performed to detect cell proliferation (*n* = 3) in the **(A)** 7.5 μg/mL control spleen-derived exosomes group. **(B)** 7.5 μg/mL YWD-treated spleen-derived exosomes group. **(C)** 30 μg/mL control spleen-derived exosomes group. **(D)** 30 μg/mL YWD-treated spleen-derived exosomes group. **(E)** 120 μg/mL control spleen-derived exosomes group, **(F)** 120 μg/mL YWD-treated spleen-derived exosomes group. **(G)** paclitaxel group. **(H)** model group (cells treated only with RPMI 1640 medium were used as the tumor cell control group). **(I)** The proliferation was determined via CCK8 assay and **(J)** tumor suppression rate. (Note: **p < 0.01 and *p < 0.05; #Independent sample *t*-test with and without YWD, indicating #*p* < 0.05).

**FIGURE 5:**
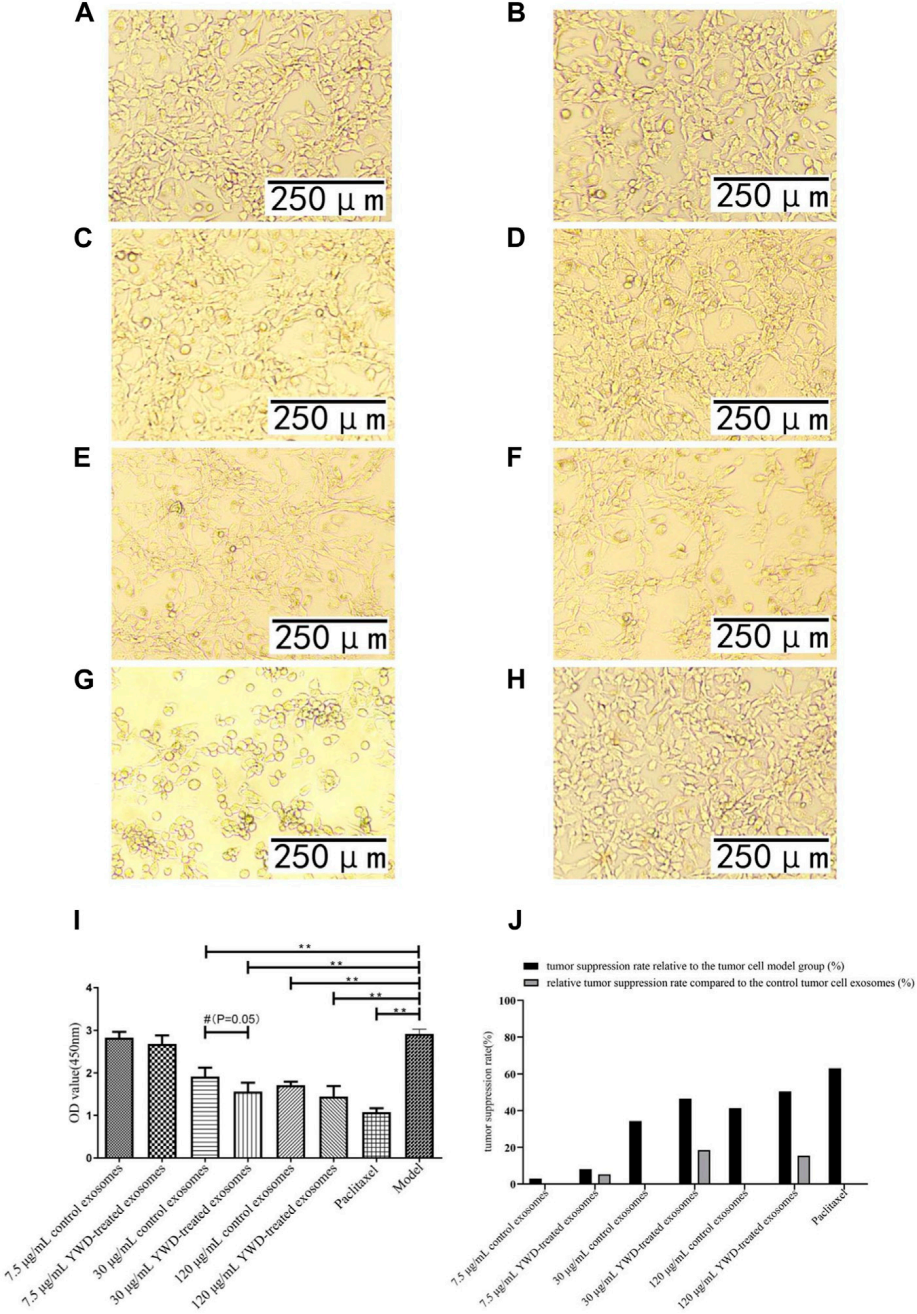
Effect of YWD-treated and control spleen-derived exosomes on the proliferation of MFC cells (×200 magnification). CCK8 assays were performed to detect cell proliferation (*n* = 4) in the **(A)** 7.5 μg/mL control spleen-derived exosomes group. **(B)** 7.5 μg/mL YWD-treated spleen-derived exosomes group. **(C)** 30 μg/mL control spleen-derived exosomes group. **(D)** 30 μg/mL YWD-treated spleen-derived exosomes group. **(E)** 120 μg/mL control spleen-derived exosomes group. **(F)** 120 μg/mL YWD-treated spleen-derived exosomes group. **(G)** paclitaxel group. **(H)** model group (cells treated only with RPMI 1640 medium were used as the tumor cell control group). **(I)** The proliferation was determined via CCK8 assay and **(J)** tumor suppression rate. (Note: **p < 0.01 and *p < 0.05; #Independent sample *t*-test with and without YWD, indicating #*p* < 0.05).

Compared to the control spleen-derived exosomes at the same concentrations, the YWD-treated spleen-derived exosomes at concentrations of 7.5 μg/mL, 30 μg/mL, and 120 μg/mL significantly decreased the HGC-27 cell viability with relative tumor inhibition rates of 14.54% (*p* < 0.01), 70.78% (*p* < 0.05), and 44.87% (*p* < 0.05), respectively (Figure 3). The best relative antitumor effect was observed after treatment of HGC-27 cells with 30 μg/mL YWD-treated spleen-derived exosomes. Compared to the control spleen-derived exosomes groups at the same concentrations, the cell viability of S180 mouse sarcoma cells after treatment with YWD-treated spleen-derived exosomes at concentrations of 7.5 μg/mL, 30 μg/mL, and 120 μg/mL was significantly lower with relative tumor inhibition rates of 38.78% (*p* < 0.05), 33.95% (*p* < 0.05), and 49.18% (*p* < 0.05), respectively (Figure 4). Compared to the control spleen-derived exosomes groups at the same concentrations, the cell viability of MFC cells after treatment with YWD-treated spleen-derived exosomes at concentrations of 7.5 μg/mL, 30 μg/mL, and 120 μg/mL was lower with relative tumor inhibition rates of 5.29%, 18.52% (*p* = 0.05), and 15.42%, respectively, but there were no significant differences (Figure 5).

As a positive control group, the paclitaxel group had a better antitumor effect in HGC-27, S180, and MFC cells than the model group.

### Colony formation assay of spleen-derived exosomes inhibiting the proliferation of HGC-27

**FIGURE 6:**
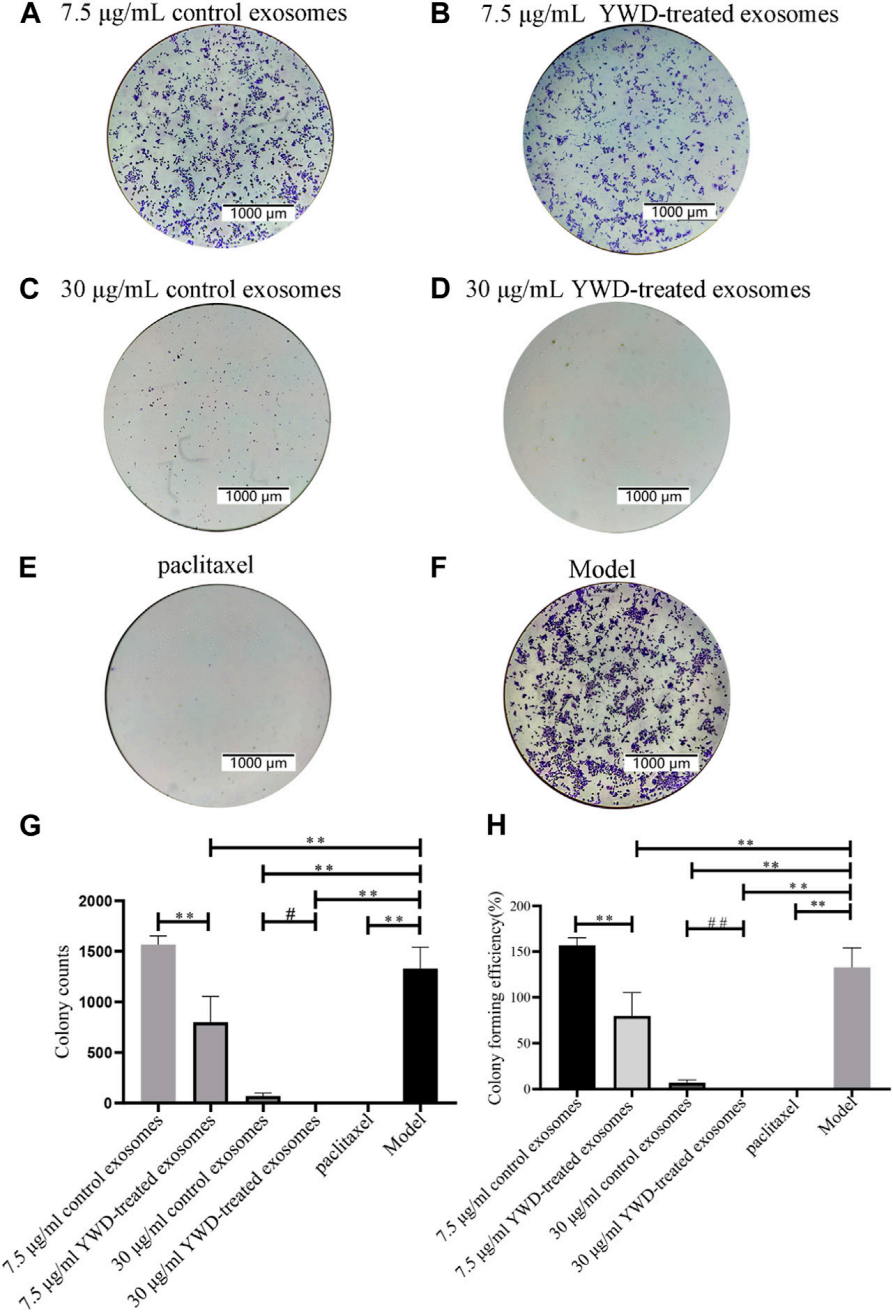
Colony formation assay of HGC-27 cells treated with spleen-derived exosomes (*n* = 3). Images of colony formation after treating HGC-27 cells with **(A)** 7.5 μg/mL control spleen-derived exosomes group, **(B)** 7.5 μg/mL YWD-treated spleen-derived exosomes group, **(C)** 30 μg/mL control spleen-derived exosomes group, **(D)** 30 μg/mL YWD-treated spleen-derived exosomes group, **(E)** paclitaxel group, and **(F)** model group (cells treated only with RPMI 1640 medium were used as the tumor cell control group), **(G)** Colony counts in each group, and **(H)** Colony forming efficiency in each group. (Note: ^**^ *p* < 0.01 and ^*^ *p* < 0.05; ^#^Independent sample *t*-test or rank sum test with and without YWD, ^##^ *p* < 0.01 and ^#^ *p* < 0.05.)

To verify whether spleen-derived exosomes after YWD treatment affect tumor proliferation, we performed a colony formation assay in HGC-27 cells (Figure 6). The average number of colonies in the 7.5 μg/mL and 30 μg/mL YWD-treated spleen-derived exosomes was 798.67 and 0.67, respectively, which was significantly less than the average number of colonies in the 7.5 μg/mL and 30 μg/mL control spleen-derived exosomes (1567.33 and 68.67, respectively; *p* < 0.01 or *p* < 0.05) with decreases of 49.04% and 99.03%, respectively. The colony forming rates of 7.5 μg/mL and 30 μg/mL YWD-treated spleen-derived exosomes were 79.87% and 0.07%, respectively, while the colony forming rates of the 7.5 μg/mL and 30 μg/mL spleen-derived control exosomes were 156.73% and 6.87%, respectively, which indicated that colony formation rates of YWD-treated spleen-derived exosomes were significantly lower compared to control spleen-derived exosomes at the same concentrations (*p* < 0.01). Thus, these results indicated that YWD-treated spleen-derived exosomes have a more significant inhibitory effect on the proliferation of HGC-27 gastric cancer cells compared to control spleen-derived exosomes.

As a positive control group, the paclitaxel group had a better antitumor effect in HGC-27 than the model group.

### YWD-treated exosomes promote apoptosis of HGC-27 cells

**FIGURE 7:**
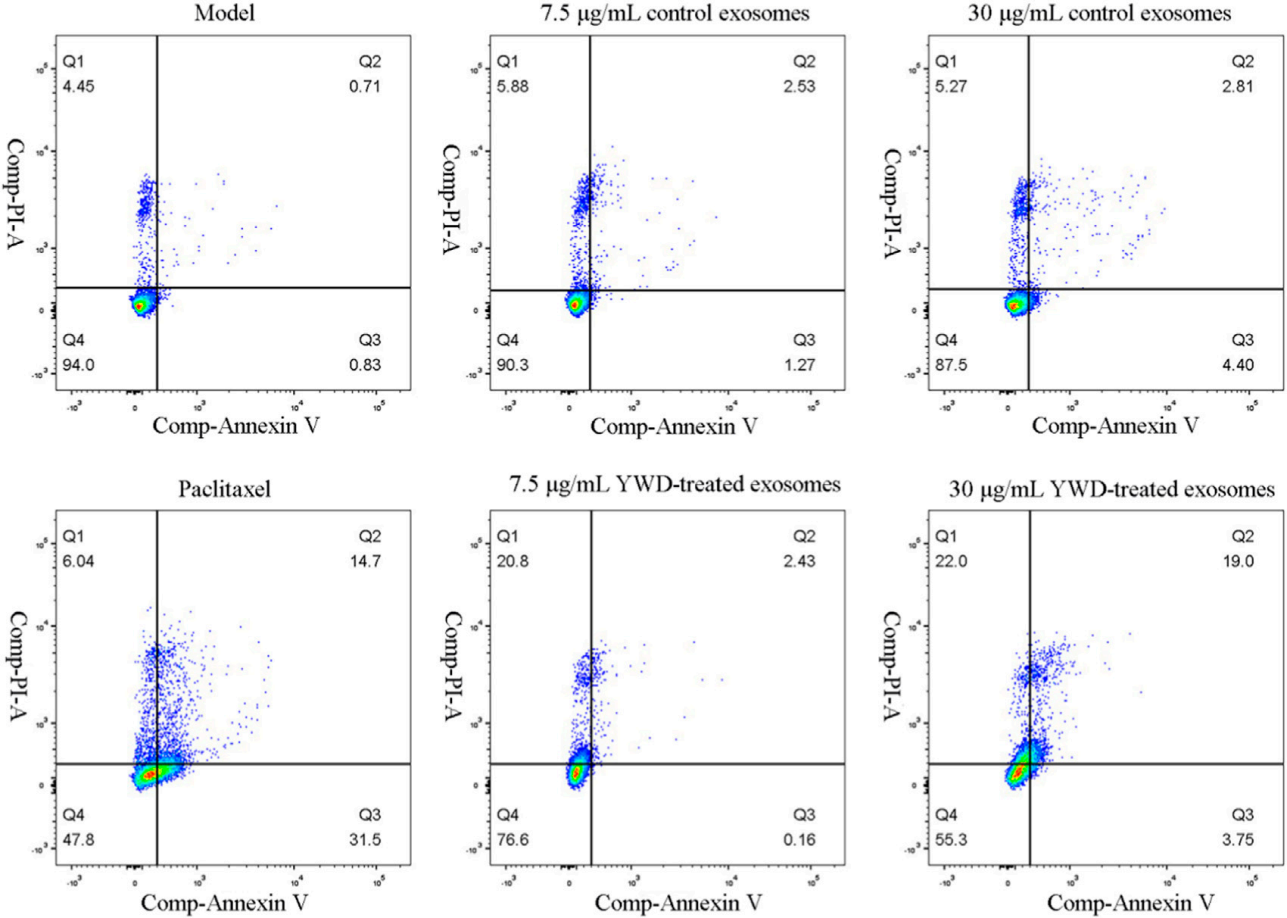
Flow cytometry results of the effect of different treatment groups on HGC-27 cell apoptosis (*n* = 3).

Flow cytometry was utilized to measure the apoptosis of HGC-27 cells after treatment with the various groups (Table 3; Figure 7). The total apoptosis rates were 11.86%, 54.81%, 20.47%, 25.91%, 29.33%, and 43.27% in the model group (cells treated only with RPMI 1640 medium were used as the tumor cell control group), 6.67 μg/mL paclitaxel group, 7.5 μg/mL control spleen-derived exosomes group, 30 μg/mL control spleen-derived exosomes group, 7.5 μg/mL YWD-treated spleen-derived exosomes group, and 30 μg/mL YWD-treated spleen-derived exosomes group, respectively. The apoptosis rate in the 30 μg/mL YWD-treated spleen-derived exosomes group was significantly higher than that in the model group and the control spleen-derived exosomes group (*p* < 0.05). In addition, the apoptosis rate in the 6.67 μg/mL paclitaxel group was significantly higher than that of the model group (*p* < 0.01). Of note, the apoptosis rate in the 120 μg/mL control spleen-derived exosomes group and the 120 μg/mL YWD-treated spleen-derived exosomes group failed to be detected by flow cytometry due to low cell proliferation.

**TABLE 3:** Flow cytometry results of the effects of different concentrations of spleen YWD-treated exosomes on HGC-27 cell apoptosis.

Group	Q1 (death, fragments)	Q2 (late apoptosis rate)	Q3 (early apoptosis rate)	Q4 (healthy/Normal)	Total apoptosis rate
Model group	8.16 ± 2.67	2.88 ± 1.60	0.82 ± 0.30	88.13 ± 4.16	11.86 ± 5.09
Paclitaxel group	30.61 ± 17.51	13.64 ± 3.03	10.56 ± 14.81	45.10 ± 1.92	54.81 ± 2.47^★^
7.5 μg/mL control spleen-derived exosomes group	16.29 ± 9.63	3.51 ± 0.73	0.67 ± 0.50	79.53 ± 9.80	20.47 ± 12.01
7.5 μg/mL YWD-treated spleen-derived exosomes group	23.79 ± 13.25	4.06 ± 0.95	1.48 ± 2.06	70.67 ± 12.13	29.33 ± 14.87
30 μg/mL control spleen-derived exosomes group	21.93 ± 2.50	3.88 ± 1.12	0.10 ± 0.05	74.07 ± 3.30	25.91 ± 4.09
30 μg/mL YWD-treated spleen-derived exosomes group	32.00 ± 7.11	9.99 ± 6.38	1.27 ± 1.75	56.77 ± 1.09	43.26 ± 1.34^☆△^

(Note: ±s; n = 3; unit = %.

☆indicates the comparison between each group and the model group, in which ★*p* < 0.01 and ☆*p* < 0.05; Δ indicates the comparison between the YWD-treated group and control group, in which ▲*p* < 0.01 and △*p* < 0.05.

(Note: The abscissa is Annexin V-FITC (FITC), which indicates early cell apoptosis; The ordinate Propidium Iodide (PI) indicates the middle and late stage of apoptosis and necrotic cells.).

## Discussion

The anticancer effects of herbal medicine have been emphasized in recent years, and some studies have shown that herbal medicine has inhibitory effects on various cancers, including intestinal cancer and liver cancer (Lee et al., 2008; Weng et al., 2010). In the theory of TCM, healthy qi is the defense system and repair system of the human body, and the fundamental cause of the occurrence and development of gastric cancer is the weakness of qi; thus, strengthening the body’s resistance and eliminating harm is the basic principle of TCM in treating cancer (Ji et al., 2016; Wu et al., 2022). In many western developed countries, complementary and alternative medicine (CAM) is advocated to manage and treat malignant tumors, and TCM is also included in CAM therapy. Increasing evidence shows that TCM has the potential to develop into a clinical treatment for various malignant tumors (Ye et al., 2015). At present, there have been many studies on the anticancer effects of single Chinese medicine and compound Chinese medicine on gastric cancer. Because TCM combined with surgical chemotherapy has more advantages for patient prognosis, it is reasonable to infer that Chinese medicine has unique antitumor advantages (Liu et al., 2017; Cao et al., 2018; Li D H et al., 2020). A TCM compound is a prescription composed of various herbs, which plays a complex and multitargeted role in clinical efficacy (Tan et al., 2022). The main function of YWD is replenishing qi and improving physical fitness, which conforms to the basic principles of TCM theory in treating cancer. According to the present study, YWD may affect the production of exosomes by multiple cell types of the spleen, thus improving tumor immunity and making the body more tumor resistant via multitargeted and multipathway effects. As the spleen is the largest secondary lymphoid organ in the body, it has a wide range of immune functions in addition to its role in hematopoiesis and erythrocyte clearance (Lewis et al., 2019). However, there is a lack of research on whether exosomes from the spleen after treatment with TCM strengthen the spleen and supplements qi, thus improving the inhibition of tumor proliferation.

In body fluids, exosomes are predominantly spherical and can be collected by sucrose pad ultracentrifugation at densities ranging from 1.13 to 1.19 g/mL; typically, exosomes exhibit a cup-like appearance under transmission electron microscopy with a diameter between 40 and 100 nm (Ning et al., 2018). CD63 is cluster of differentiation 63 (CD63) or lysosome-associated membrane protein 3, is a member of the family of four transmembrane proteins that are commonly expressed on exosomes and are characteristic of exosomes (Jeppesen et al., 2019). CD63 is involved in signal transduction in various types of immune cells, and it contributes to immune metabolism (Im et al., 2021). In addition, TSG101, ALIX, and ALG-2 are important markers of exosomes (Inuzuka et al., 2013; Gurunathan et al., 2019; Hoshino et al., 2020). In the present study, western blot analysis indicated that the rat spleen-derived exosomes expressed CD63, TGS101, ALIX, and ALG-2.

Exosomes have a vesicular structure composed of cell membrane-like structures, and PHK67 is one of the markers of cell membranes, indicating that exosomes can also be recognized by PHK-67 staining (Li et al., 2015). In the present study, exosomes isolated from spleen tissue had high PHK67 protein expression levels. In addition, the cytoplasm of HGC-27 cells had high levels of PHK67 protein after treating cells with exosomes, indicating that exosomes isolated from spleen tissues were taken up by HGC-27 cells.

Several roles of exosomes have been reported in the literature, including inhibiting cell proliferation, differentiation, and invasion as well as inducing apoptosis. Human umbilical cord blood mesenchymal stromal cell-derived exosomes have been used to deliver anti-miRNA oligonucleotides, which are specifically taken up by tumor cells to exert antitumor functions (Han et al., 2021). Neutrophils are the most abundant innate immune cells in the human circulation, and exosomes from neutrophils induce apoptosis by delivering cytotoxic proteins and activating the cysteine aspartate signaling pathway in HGC-27 cells, human HepG2 hepatocellular carcinoma cells, and human SW480 colon cancer cells (Zhang et al., 2022). Exosomes carrying chimeric antigen receptor (CAR)-expressing significantly inhibit the growth of endogenous and exogenous mesothelin (MSLN)-positive triple-negative breast cancer (TNBC) cells (Yang P et al., 2021). In the present study, treatment with YWD-treated spleen-derived exosomes inhibited the proliferation of HGC-27 and S180 cells as shown by the CCK8 assay, indicating that these cells are sensitive to YWD-treated spleen-derived exosomes. And it showed that compared with S180 cells, HGC-27 cells are the most sensitive to YWD-treated spleen-derived exosomes.

Colony forming efficiency (CFE) is a measure of the viability of a cell group formed by the progenies of a single cell that proliferates for more than six generations *in vitro* (Xu et al., 2019). The colony formation assay of HGC-27 proliferation inhibition by YWD-treated spleen-derived exosomes showed that the number of colonies decreased by 49.04% in the 7.5 μg/mL group and by 99.03% in the 30 μg/mL group. The colony formation rate of the 30 μg/mL YWD-treated spleen-derived exosomes group (0.07%) was significantly lower than that of the 30 μg/mL control spleen-derived exosomes group (6.87%). These results indicated that YWD-treated spleen-derived exosomes inhibit the colony formation of HGC-27 cells in a dose-dependent manner.

Because a distinctive feature of tumors is apoptosis resistance (Carneiro and El-Deiry, 2020), induction of apoptosis is an important direction in antitumor research (Shimbo et al., 2014; Lou et al., 2015). Annexin V-FITC/PI staining is a commonly used flow cytometry apoptosis assay. PI is a nucleic acid dye that does not penetrate intact cell membranes, but it penetrates the cell membrane in cells with advanced apoptosis and necrotic cells. In healthy cells, phosphatidylserine (PS) is only distributed on the inner side of lipid bilayer of cell membrane, while in early apoptosis, PS in the cell membrane flips from the inner side to the outside of the lipid membrane. PS ectopia occurs before nuclear rupture, DNA fragmentation, and the appearance of apoptosis-associated proteins, rendering Annexin V binding to PS an important marker event in the early stages of apoptosis (Chen et al., 2020). In the present study, flow cytometry analysis indicated that the number of Annexin V+/PI + HGC-27 cells was increased in the YWD-treated spleen-derived exosomes groups, indicating that the apoptosis rate significantly increased with increasing concentrations of YWD-treated exosomes.

Chinese herbal medicine is traditionally used to prevent and treat many diseases in China, especially autoimmune diseases and tumors. Studies have suggested that *Astragalus membranaceus* (11.8%) is the most frequently used single medicine in the combination of TCM with chemotherapy for cancer treatment (Shih et al., 2021), which is consistent with the monarch drug of YWD. Previous studies have reported that *A. membranaceus* has a potential immunomodulatory effect when used in combination with chemotherapy and radiotherapy because it promotes the activity of lymphocytes, natural killer (NK) cells, and macrophages. *Astragalus membranaceus* has been demonstrated to increase the secretion of interferon and tumor necrosis factor (TNF) as well as activate lymphocytes, NK cells, and macrophages to resist tumors. In addition, *A. membranaceus* cooperates with IL-2 to stimulate NK cells activated by lymphokines to attack tumor cells (Yoshida et al., 1997; No author listed, 2003). In addition, Astragalus polysaccharide (APS) modulates host organism immunity by activating the TLR4-mediated MyD88-dependent signaling pathway to enhance immunity and exert antitumor effects (Zhou et al., 2017). The petroleum ether fraction (PEF) of trichothecene triggers extrinsic and intrinsic apoptotic pathways, and it increases oxidative stress in cervical cancer HeLa cells, thereby inhibiting tumor cell viability (Xiong et al., 2015). *Radix Ophiopogonis* inhibits transforming growth factor beta 1 (TGF-β1)-mediated metastatic behavior of MDA-MB-231 cells by regulating the integrin subunit beta 1 (ITGB1)/focal adhesion kinase (FAK)/Src/AKT/β-catenin signaling axis (Zhu et al., 2020). In addition, exosomes contain immune-related molecular substances, such as FasL, tumor necrosis factor-related apoptosis-inducing ligand (TRAIL)-related TNF family proteins, and transmembrane proteins (Stenqvist et al., 2013; Cheng et al., 2021), which may be related to their antitumor effects. However, the specific mechanisms require further investigation.

Given the complex ingredients of the formula, it is difficult to determine its exact mechanism of action, which is one of the limitations of this study: Chinese herbal compound contains many potentially useful components, so it is difficult to understand the metabolism process and anti-tumor mechanism *in vivo* as clearly as the chemical monomer.

In general, the anti-tumor effect was improved by the influence of spleen exosomes, which has been reported in this paper, but the clearer mechanism needs to be designed and carried out in the next study, such as the overall related experiments of exosomes entering tumor bodies, and the effects on tumor growth metabolism and immunohistochemistry.

In the present study, both YWD-treated and control group of spleen-derived exosomes could enter tumor cells when co-cultured with tumor cells; YWD-treated spleen-derived exosomes had antiproliferative effects on MFC, S180, and HGC-27 tumor cells *in vitro*, with HGC-27 cells showing the most sensitive response. The spleen-derived exosomes of rats treated with YWD have more significant anti-tumor effect on tumor cells than the same exosomes without YWD; The mechanism of spleen-derived exosomes inhibiting the proliferation of HGC-27 cells may be related to enhanced cell apoptosis. We intend to further investigate different molecular signals of these exosomes and their specific mechanisms of action in the future.

## Conclusion

The present study investigated the effect of YWD on the proliferation and apoptosis of HGC-27 gastric cancer cells via spleen-derived exosomes. The results showed that the YWD-treated spleen-derived exosomes inhibited HGC-27 cell proliferation but induced HGC-27 cell apoptosis. Flow cytometry analysis showed that the total apoptosis rate of the experimental group was 43.27%, which was significantly higher than that of the control group (25.91%). The CCK8 assay confirmed that, compared to the control spleen-derived exosomes group, the relative tumor inhibition rate was 70.78% in the YWD-treated spleen-derived exosomes group. Colony formation assay indicated that compared with the control group, the YWD-treated spleen-derived exosomes group’s colony lumps have reduced by 99.03%. These findings indicated that YWD inhibits the growth of gastric cancer cells through spleen-derived exosomes. These results provide a basis for the development of Chinese medicine therapy for clinical application in gastric cancer treatment.

## Data Availability

The original contributions presented in the study are included in the article/Supplementary Material, further inquiries can be directed to the corresponding authors.

## Abbreviations

HGC-27 cells, human gastric cancer-27 cells; MFC cells, Murine forestomach carcinoma; S180 cells, mouse malignant sarcoma cells; YWD, Yiwei decoction; TCM, traditional Chinese medicine.

## References

[B1] Astragalus membranaceus (2003). Astragalus membranaceus. Monogr. Altern. Med. Rev. 8, 72–77. 12611564

[B2] Besse B. Charrier M. Lapierre V. Dansin E. Lantz O. Planchard D. (2016). Dendritic cell-derived exosomes as maintenance immunotherapy after first line chemotherapy in nsclc. Oncoimmunology 5, e1071008. 10.1080/2162402X.2015.1071008 27141373 PMC4839329

[B3] Cao B. Yu S. Y. Zhu Z. Y. Jiang X. X. Zhou Q. H. Hou K. (2018). Antitumor effects of yi-wei-jie-du decoction (ywjd) against gastric carcinoma via bgc-803 tumor xenografts mice model. Semantic Sch.

[B5] Carneiro B. A. El-Deiry W. S. (2020). Targeting apoptosis in cancer therapy. Nat. Rev. Clin. Oncol. 17, 395–417. 10.1038/s41571-020-0341-y 32203277 PMC8211386

[B6] Chen J. Chen J. Cheng Y. Fu Y. Zhao H. Tang M. (2020). Mesenchymal stem cell-derived exosomes protect beta cells against hypoxia-induced apoptosis via mir-21 by alleviating er stress and inhibiting p38 mapk phosphorylation. Stem Cell Res. Ther. 11, 97. 10.1186/s13287-020-01610-0 32127037 PMC7055095

[B7] Cheng Q. Dai Z. Shi X. Duan X. Wang Y. Hou T. (2021). Expanding the toolbox of exosome-based modulators of cell functions. Biomaterials 277, 121129. 10.1016/j.biomaterials.2021.121129 34534861 PMC8501890

[B8] Chi M. Liu J. Mei C. Shi Y. Liu N. Jiang X. (2022). Tead4 functions as a prognostic biomarker and triggers emt via pi3k/akt pathway in bladder cancer. J. Exp. Clin. Cancer Res. 41, 175. 10.1186/s13046-022-02377-3 35581606 PMC9112458

[B9] Dong Y. Ding D. Gu J. Chen M. Li S. (2022). Alpha-2 heremans schmid glycoprotein (ahsg) promotes the proliferation of bladder cancer cells by regulating the tgf-beta signalling pathway. Bioengineered 13, 14282–14298. 10.1080/21655979.2022.2081465 35746836 PMC9342194

[B10] Dong Y. Zhao L. S. Wu R. Z. Chen W. Chai K. Q. (2020). Study on the traditional Chinese medicine serum pharmacochemistry of yiwei decoction. World Sci. Technology-Modernization Traditional Chin. Med. 22, 84–92. 10.11842/wst.20190401005

[B11] Fu M. Gu J. Jiang P. Qian H. Xu W. Zhang X. (2019). Exosomes in gastric cancer: Roles, mechanisms, and applications. Mol. Cancer 18, 41. 10.1186/s12943-019-1001-7 30876419 PMC6419325

[B12] Gullo I. Grillo F. Mastracci L. Vanoli A. Carneiro F. Saragoni L. (2020). Precancerous lesions of the stomach, gastric cancer and hereditary gastric cancer syndromes. Pathologica 112, 166–185. 10.32074/1591-951X-166 33179620 PMC7931572

[B13] Gurunathan S. Kang M. H. Jeyaraj M. Qasim M. Kim J. H. (2019). Review of the isolation, characterization, biological function, and multifarious therapeutic approaches of exosomes. Cells 8. 10.3390/cells8040307 PMC6523673 30987213

[B14] Han S. Li G. Jia M. Zhao Y. He C. Huang M. (2021). Delivery of anti-mirna-221 for colorectal carcinoma therapy using modified cord blood mesenchymal stem cells-derived exosomes. Front. Mol. Biosci. 8, 743013. 10.3389/fmolb.2021.743013 34616773 PMC8488275

[B15] Hoshino A. Kim H. S. Bojmar L. Gyan K. E. Cioffi M. Hernandez J. (2020). Extracellular vesicle and particle biomarkers define multiple human cancers. Cell 182, 1044–1061. 10.1016/j.cell.2020.07.009 32795414 PMC7522766

[B16] Huang C. S. Ho J. Y. Chiang J. H. Yu C. P. Yu D. S. (2020). Exosome-derived linc00960 and linc02470 promote the epithelial-mesenchymal transition and aggressiveness of bladder cancer cells. Cells 9. 10.3390/cells9061419 PMC7349410 32517366

[B17] Im Y. Yoo H. Ko R. E. Lee J. Y. Park J. Jeon K. (2021). Exosomal cd63 in critically ill patients with sepsis. Sci. Rep. 11, 20300. 10.1038/s41598-021-99777-w 34645935 PMC8514522

[B18] Inuzuka T. Inokawa A. Chen C. Kizu K. Narita H. Shibata H. (2013). Alg-2-interacting tubby-like protein superfamily member plscr3 is secreted by an exosomal pathway and taken up by recipient cultured cells. Biosci. Rep. 33. 10.1042/BSR20120123 PMC3590573 23350699

[B19] Jeppesen D. K. Fenix A. M. Franklin J. L. Higginbotham J. N. Zhang Q. Zimmerman L. J. (2019). Reassessment of exosome composition. Cell 177, 428–445. 10.1016/j.cell.2019.02.029 30951670 PMC6664447

[B20] Ji Q. Luo Y. Q. Wang W. H. Liu X. Li Q. Su S. B. (2016). Research advances in traditional Chinese medicine syndromes in cancer patients. J. Integr. Med. 14, 12–21. 10.1016/S2095-4964(16)60237-6 26778224

[B21] Jia J. Guo S. Zhang D. Tian X. Xie X. (2020). Exosomal-lncRNA dleu1 accelerates the proliferation, migration, and invasion of endometrial carcinoma cells by regulating microRNA-e2f3. Onco Targets Ther. 13, 8651–8663. 10.2147/OTT.S262661 32904666 PMC7457553

[B22] Johnston F. M. Beckman M. (2019). Updates on management of gastric cancer. Curr. Oncol. Rep. 21, 67. 10.1007/s11912-019-0820-4 31236716

[B23] Lee S. H. Cekanova M. Baek S. J. (2008). Multiple mechanisms are involved in 6-gingerol-induced cell growth arrest and apoptosis in human colorectal cancer cells. Mol. Carcinog. 47, 197–208. 10.1002/mc.20374 18058799 PMC2430145

[B24] Lewis S. M. Williams A. Eisenbarth S. C. (2019). Structure and function of the immune system in the spleen. Sci. Immunol. 4. 10.1126/sciimmunol.aau6085 PMC6495537 30824527

[B25] Li D. H. Su Y. F. Sun C. X. Fan H. F. (2020). Effect of shunqi yiwei decoction combined with sox chemotherapy on ad-vanced gastric cancer. Acta Medica Mediterr., 36–2313. 10.19193‬/0393-6384‬_2020_4_360

[B26] Li X. Li C. Zhang L. Wu M. Cao K. Jiang F. (2020). The significance of exosomes in the development and treatment of hepatocellular carcinoma. Mol. Cancer 19, 1. 10.1186/s12943-019-1085-0 31901224 PMC6942270

[B27] Li Y. J. Diao Z. Y. Xue P. P. Shen L. Gong P. Yan G. J. (2015). Isolation and identification of placental exosomes from maternal serum. J. Med. Postgraduates, 632–636.

[B28] Lin X. Han T. Xia Q. Cui J. Zhuo M. Liang Y. (2021). Chpf promotes gastric cancer tumorigenesis through the activation of e2f1. Cell Death Dis. 12, 876. 10.1038/s41419-021-04148-y 34564711 PMC8464597

[B29] Liu X. Xiu L. J. Jiao J. P. Zhao J. Zhao Y. Lu Y. (2017). Traditional Chinese medicine integrated with chemotherapy for stage iv non-surgical gastric cancer: A retrospective clinical analysis. J. Integr. Med. 15, 469–475. 10.1016/S2095-4964(17)60377-7 29103417

[B30] Lou G. Song X. Yang F. Wu S. Wang J. Chen Z. (2015). Exosomes derived from mir-122-modified adipose tissue-derived mscs increase chemosensitivity of hepatocellular carcinoma. J. Hematol. Oncol. 8, 122. 10.1186/s13045-015-0220-7 26514126 PMC4627430

[B31] Mahul-Mellier A. Strappazzon F. Chatellard-Causse C. Blot B. Béal D. Torch S. (2009). Alix and alg-2 make a link between endosomes and neuronal death. Biochem. Soc. Trans. 37, 200–203. 10.1042/BST0370200 19143631

[B32] Ning X. Zhang H. Wang C. Song X. (2018). Exosomes released by gastric cancer cells induce transition of pericytes into cancer-associated fibroblasts. Med. Sci. Monit. 24, 2350–2359. 10.12659/msm.906641 29668670 PMC5922989

[B33] Qiu H. Cao S. Xu R. (2021). Cancer incidence, mortality, and burden in China: A time-trend analysis and comparison with the United States and United Kingdom based on the global epidemiological data released in 2020. Cancer Commun. (Lond) 41, 1037–1048. 10.1002/cac2.12197 34288593 PMC8504144

[B34] Qiu W. W. Chen Q. Y. Zheng W. Z. He Q. C. Huang Z. N. Xie J. W. (2022). Postoperative follow-up for gastric cancer needs to be individualized according to age, tumour recurrence pattern, and recurrence time. Eur. J. Surg. Oncol. 48, 1790–1798. 10.1016/j.ejso.2022.02.025 35279349

[B35] Rashid M. H. Borin T. F. Ara R. Piranlioglu R. Achyut B. R. Korkaya H. (2021). Critical immunosuppressive effect of mdsc-derived exosomes in the tumor microenvironment. Oncol. Rep. 45, 1171–1181. 10.3892/or.2021.7936 33469683 PMC7860000

[B36] Shi X. Cheng Q. Hou T. Han M. Smbatyan G. Lang J. E. (2020). Genetically engineered cell-derived nanoparticles for targeted breast cancer immunotherapy. Mol. Ther. 28, 536–547. 10.1016/j.ymthe.2019.11.020 31843452 PMC7001084

[B37] Shih W. T. Yang P. R. Shen Y. C. Yang Y. H. Wu C. Y. (2021). Traditional Chinese medicine enhances survival in patients with gastric cancer after surgery and adjuvant chemotherapy in taiwan: A nationwide matched cohort study. Evid. Based Complement. Altern. Med. 2021, 7584631. 10.1155/2021/7584631 PMC7889357 33628314

[B38] Shimbo K. Miyaki S. Ishitobi H. Kato Y. Kubo T. Shimose S. (2014). Exosome-formed synthetic microrna-143 is transferred to osteosarcoma cells and inhibits their migration. Biochem. Biophys. Res. Commun. 445, 381–387. 10.1016/j.bbrc.2014.02.007 24525123

[B39] Smyth E. C. Nilsson M. Grabsch H. I. van Grieken N. C. Lordick F. (2020). Gastric cancer. Lancet 396, 635–648. 10.1016/S0140-6736(20)31288-5 32861308

[B40] Stenqvist A. C. Nagaeva O. Baranov V. Mincheva-Nilsson L. (2013). Exosomes secreted by human placenta carry functional fas ligand and trail molecules and convey apoptosis in activated immune cells, suggesting exosome-mediated immune privilege of the fetus. J. Immunol. 191, 5515–5523. 10.4049/jimmunol.1301885 24184557

[B41] Sung H. Ferlay J. Siegel R. L. Laversanne M. Soerjomataram I. Jemal A. (2021). Global cancer statistics 2020: Globocan estimates of incidence and mortality worldwide for 36 cancers in 185 countries. Ca Cancer J. Clin. 71, 209–249. 10.3322/caac.21660 33538338

[B42] Tan Y. Wang H. Xu B. Zhang X. Zhu G. Ge Y. (2022). Chinese herbal medicine combined with oxaliplatin-based chemotherapy for advanced gastric cancer: A systematic review and meta-analysis of contributions of specific medicinal materials to tumor response. Front. Pharmacol. 13, 977708. 10.3389/fphar.2022.977708 36091754 PMC9453215

[B43] Thery C. Witwer K. W. Aikawa E. Alcaraz M. J. Anderson J. D. Andriantsitohaina R. (2018). Minimal information for studies of extracellular vesicles 2018 (misev2018): A position statement of the international society for extracellular vesicles and update of the misev2014 guidelines. J. Extracell. Vesicles 7, 1535750. 10.1080/20013078.2018.1535750 30637094 PMC6322352

[B44] Viaud S. Terme M. Flament C. Taieb J. Andre F. Novault S. (2009). Dendritic cell-derived exosomes promote natural killer cell activation and proliferation: A role for nkg2d ligands and il-15ralpha. Plos One 4, e4942. 10.1371/journal.pone.0004942 19319200 PMC2657211

[B45] Villatoro A. J. Martin-Astorga M. Alcoholado C. Becerra J. (2020). Canine colostrum exosomes: Characterization and influence on the canine mesenchymal stem cell secretory profile and fibroblast anti-oxidative capacity. Bmc Vet. Res. 16, 417. 10.1186/s12917-020-02623-w 33138803 PMC7607682

[B46] Wang M. Zhao C. Shi H. Zhang B. Zhang L. Zhang X. (2014). Deregulated micrornas in gastric cancer tissue-derived mesenchymal stem cells: Novel biomarkers and a mechanism for gastric cancer. Br. J. Cancer 110, 1199–1210. 10.1038/bjc.2014.14 24473397 PMC3950864

[B47] Wang Y. Li P. Mao S. Mo Z. Cao Z. Luo J. (2021). Exosome ctla-4 regulates pten/cd44 signal pathway in spleen deficiency internal environment to promote invasion and metastasis of hepatocellular carcinoma. Front. Pharmacol. 12, 757194. 10.3389/fphar.2021.757194 34744733 PMC8564353

[B48] Weng C. J. Wu C. F. Huang H. W. Ho C. T. Yen G. C. (2010). Anti-invasion effects of 6-shogaol and 6-gingerol, two active components in ginger, on human hepatocarcinoma cells. Mol. Nutr. Food Res. 54, 1618–1627. 10.1002/mnfr.201000108 20521273

[B49] Wu H. Y. Miao X. M. Liu Y. Zhang S. Li C. Hao J. (2022). Clinical efficacy of modified yiwei shengyang decoction combined with folfox4 chemotherapy regimen in the treatment of advanced gastric cancer and its effect on tumor marker levels. Evid. Based Complement. Altern. Med. 2022, 6234032. 10.1155/2022/6234032 PMC9098293 35571732

[B50] Xiong Y. Wu X. Rao L. (2015). Tetrastigma hemsleyanum (sanyeqing) root tuber extracts induces apoptosis in human cervical carcinoma hela cells. J. Ethnopharmacol. 165, 46–53. 10.1016/j.jep.2015.02.030 25701754

[B51] Xu J. Xiao Y. Liu B. Pan S. Liu Q. Shan Y. (2020). Exosomal malat1 sponges mir-26a/26b to promote the invasion and metastasis of colorectal cancer via fut4 enhanced fucosylation and pi3k/akt pathway. J. Exp. Clin. Cancer Res. 39, 54. 10.1186/s13046-020-01562-6 32209115 PMC7092616

[B52] Xu Y. Y. Han Y. Tian L. Q. Zhou J. Wei C. Zhang D. D.(2019). Comparison of several *in vitro* cytotoxicity assays in GB/T 16886.5—2017. Chin. Med. Equip. J. 40, 74–77. 10.19745/j.1003-8868.2019174

[B53] Yang J. Luo X. L. Zhu L. Liu J. M. Xu Y. F. (2021). Network meta-analysis of 10 kinds of oral Chinese patent medicine combined with chemotherapy in treatment of gastric cancer. Chin. J. Exp. traditional Med. formulae 27, 181–195. 10.13422/j.cnki.syfjx.20202118

[B54] Yang P. Cao X. Cai H. Feng P. Chen X. Zhu Y. (2021). The exosomes derived from car-t cell efficiently target mesothelin and reduce triple-negative breast cancer growth. Cell Immunol. 360, 104262. 10.1016/j.cellimm.2020.104262 33373818

[B55] Ye L. Jia Y. Ji K. E. Sanders A. J. Xue K. Ji J. (2015). Traditional Chinese medicine in the prevention and treatment of cancer and cancer metastasis. Oncol. Lett. 10, 1240–1250. 10.3892/ol.2015.3459 26622657 PMC4533180

[B56] Yoshida Y. Wang M. Q. Liu J. N. Shan B. E. Yamashita U. (1997). Immunomodulating activity of Chinese medicinal herbs and oldenlandia diffusa in particular. Int. J. Immunopharmacol. 19, 359–370. 10.1016/s0192-0561(97)00076-3 9568540

[B57] Zhang F. Li R. Yang Y. Shi C. Shen Y. Lu C. (2019). Specific decrease in b-cell-derived extracellular vesicles enhances post-chemotherapeutic cd8(+) t cell responses. Immunity 50, 738–750. 10.1016/j.immuni.2019.01.010 30770248

[B58] Zhang J. Ji C. Zhang H. Shi H. Mao F. Qian H. (2022). Engineered neutrophil-derived exosome-like vesicles for targeted cancer therapy. Sci. Adv. 8, j8207. 10.1126/sciadv.abj8207 PMC8754405 35020437

[B59] Zhang L. Yu D. (2019). Exosomes in cancer development, metastasis, and immunity. Biochim. Biophys. Acta Rev. Cancer 1871, 455–468. 10.1016/j.bbcan.2019.04.004 31047959 PMC6542596

[B60] Zhang Y. Bi J. Huang J. Tang Y. Du S. Li P. (2020). Exosome: A review of its classification, isolation techniques, storage,diagnostic and targeted therapy applications. Int. J. Nanomedicine 15, 6917–6934. 10.2147/IJN.S264498 33061359 PMC7519827

[B61] Zhou L. Liu Z. Wang Z. Yu S. Long T. Zhou X. (2017). Astragalus polysaccharides exerts immunomodulatory effects via tlr4-mediated myd88-dependent signaling pathway *in vitro* and *in vivo* . Sci. Rep. 7, 44822. 10.1038/srep44822 28303957 PMC5355992

[B62] Zhou W. Zhou Y. Chen X. Ning T. Chen H. Guo Q. (2021). Pancreatic cancer-targeting exosomes for enhancing immunotherapy and reprogramming tumor microenvironment. Biomaterials 268, 120546. 10.1016/j.biomaterials.2020.120546 33253966

[B63] Zhu X. Wang K. Chen Y. (2020). Ophiopogonin d suppresses tgf-beta1-mediated metastatic behavior of mda-mb-231 breast carcinoma cells via regulating itgb1/fak/src/akt/beta-catenin/mmp-9 signaling axis. Toxicol Vitro 69, 104973. 10.1016/j.tiv.2020.104973 32818624

